# F20. SEX-SPECIFIC STRUCTURAL AND FUNCTIONAL CIRCUIT DIFFERENCES IN YOUTH WITH PSYCHOSIS SPECTRUM SYMPTOMS

**DOI:** 10.1093/schbul/sby017.551

**Published:** 2018-04-01

**Authors:** Grace Jacobs, Stephanie Ameis, Joseph Viviano, Erin Dickie, Anne Wheeler, Sonja Stojanovski, Aristotle Voineskos

**Affiliations:** 1 University of Toronto, CAMH; 2 CAMH; 3 University of Toronto, Hospital for Sick Children

## Abstract

**Background:**

Functional connectivity differences in the cortico-thalamic-striatal-cortical (CTSC) circuit, as well as altered subcortical region volumes have been observed in schizophrenia. In this study, structural and functional magnetic resonance imaging (MRI) were used in a large child and youth sample aged 11–21 years (n=1134) including children with psychosis spectrum (PS) symptoms (n=312) to further understanding of these biomarkers in youth outside of high risk groups and with a wider range of symptom severity.

**Methods:**

Structural subregions of the thalamus and striatum were identified using the segmentation tool MAGeT Brain. Functional subregions were segmented based on functional connectivity with the 7 functional networks identified in Yeo et al, 2011. Average time series from functional subregions were correlated vertex-wide with cortical surfaces and Fisher Z transformed. FSL’s PALM was used to examine differences and interactions between PS groups and sex. Age and in scanner motion (mean framewise displacement) were covaried for and a family wise error rate correction was applied. Structural subregion volume differences and interactions between PS groups and sex were investigated statistically using analyses of covariance (ANCOVA) with a false discovery rate of 5% correction for multiple testing. Age, intracranial volume, WRAT score and current medication use were covaried for.

**Results:**

Sex-specific differences between PS and non-PS youth in structural subregion volumes were seen in both the striatum and thalamus. There was a persistent pattern of increased volumes in girls with PS symptoms, but decreased volumes in boys with PS symptoms compared to non-PS youth in the bilateral posterior putamen of the striatum (F=9.26, pFDR=0.006), higher order thalamic bilateral pulvinar (F=9.85, pFDR=0.004), left medial dorsal nuclei (F=7.42, pFDR=0.01), as well as first order thalamic left ventral posterior nucleus (F=6.47, pFDR=0.02), medial geniculate nucleus (F=10.03, pFDR=0.004) and bilateral lateral geniculate nuclei (F=5.7, pFDR=0.03). However, both PS girls and boys had increased nucleus accumbens volumes (t=2.66, pFDR=0.02). Decreased functional connectivity was found in PS youth between a striatal subregion in the right posterior putamen (corresponding to the dorsal attention network) and occipital areas (pFWE=0.005). This pattern was found to be driven by differences in specifically PS boys and not PS girls (pFWE=0.004).

**Discussion:**

Multiple sex-specific structural differences between PS and non-PS youth were found in striatal and thalamic subregions. Hypo-connectivity between the striatal posterior putamen and occipital regions in PS boys overlap with structural increases in this subcortical volume in PS boys. Finding these early indicators is a key strategy to provide insight into neural mechanisms underlying the development of psychosis with the aim to improve and better target treatments.

